# Assessment of the Perception and Worries of Saudi Healthcare Providers About the Application of Artificial Intelligence in Saudi Health Facilities

**DOI:** 10.7759/cureus.42858

**Published:** 2023-08-02

**Authors:** Marwa Elnaggar, Zaid A Alharbi, Aasheq M Alanazi, Saleh O Alsaiari, Abdullah M Alhemaidani, Sami F Alanazi, Muteb M Alanazi

**Affiliations:** 1 Department of Community and Family Medicine, College of Medicine, Jouf University, Sakakah, SAU; 2 Department of Medical Education, College of Medicine, Suez Canal University, Ismailia, EGY; 3 College of Medicine, Jouf University, Sakakah, SAU

**Keywords:** saudi arabia, perception, healthcare workers, artificial intelligence, ai

## Abstract

Objective

This study is aimed at assessing the perception and worries of Saudi healthcare providers about the application of artificial intelligence (AI) in Saudi healthcare facilities.

Methods

The study adopted a cross-sectional study involving 1026 Saudi healthcare providers between January 2023 and April 2023. The target population was healthcare providers across Saudi health facilities. Online questionnaires were administered through social media platforms. Data were analyzed using SPSS Statistics, version 26.0 (IBM Corp., Armonk, NY) to obtain important insights.

Results

The results of this study indicated that more than half (55.2%) of the respondents had good knowledge of AI, with (48.1%) of them being familiar with the application of AI in their specialty. A good proportion of the participants (57.9%) knew at least one term about the difference between machine learning and deep learning. More than half (69.9%) of the participants indicated that they had at one point in time used speech recognition or transcription application in their work. A large section (73.3%) of healthcare providers believed that AI would replace them at their job. A vast majority (84.9%) of the participants agreed that collaboration between medical schools with engineering and computer science faculties could be a game changer to provide a road for incorporating AI into medical curricula. The mean perception of AI in this study was 37.6 (SD=8.41; range 0-241). Age, level of health, health profession, and working experience all significantly impacted the positive perception score (p=0.021; p=0.031; p=0.041; p=0.026). However, there was no significant association between gender, nationality, and Saudi regions with a mean positive perception score.

Conclusion

There was a positive perception of AI among Saudi healthcare providers. Even though a substantial majority of Saudi healthcare providers were worried that AI would replace their jobs, the study revealed that AI serves as a crucial practitioner’s tool rather than a physician’s replacement.

## Introduction

Computer science's artificial intelligence (AI) field can understand complex medical data. Its ability to find significant relationships in data collection can be employed in various clinical situations for diagnosis, therapy, and outcome prediction [1]. AI is described as the ability of a digital computer to perform tasks commonly associated with intelligent beings [2]. The subject of AI has experienced magnificent development and growth over the past two decades. AI has gained amazing applications in various fields, including medicine. AI is swiftly gaining the consideration of researchers worldwide [3]. AI will eventually substantially impact patient care. 

AI, which mimics human cognitive functions, is a forward leap in innovation and has fascinated the minds of scientists all over the globe. Healthcare providers can use AI to guarantee better results, quality therapy, and accomplish accuracy. AI can assist with anticipating the letdowns in clinical scenarios and illustrate dependable solutions [3]. With medical AI, healthcare organizations already utilize AI's ability to fundamentally alter technology. Radiological image analysis with AI support uses deep learning. This machine uses neural networks to learn from unstructured data to identify disease patterns that may be undetected by experts. This application has had tremendous success in patient care and medical practice [4]. Although most health experts acknowledge the value of AI in the medical area, many are concerned about the possible negative effects of its extensive usage in clinical practice [5]. This demonstrates the need for improved instruction and transparent regulatory regimes [6]. Health issues and hospital visits have grown significantly over the last few years, which has led to a steady increase in patient data. Clinical decision support systems (CDSS) are becoming one of the most important instruments for healthcare professionals and hospital administration. These automated systems analyze patient data using prior clinical knowledge to help medical practitioners, including dental professionals make clinical judgments regarding the diagnosis and treatment of diseases [7].

Although most health professionals acknowledge the value of AI in the medical area, there should be processes in place to regulate the caliber of the algorithms and systems employed in AI. Furthermore, there are safety concerns with AI systems. Ambiguous responsibility in its use is another difficulty in using AI. The liability for any unintended repercussions patients may suffer because of adverse events in the AI system and algorithm usage is frequently unclear. Replacing humans with autonomous agents opens several ethical and legal challenges that threaten the current legal system [8,9] To the best of our knowledge, this is one of the first studies in Saudi Arabia that measures the perception and worries of healthcare providers (including medical doctors, dentists, nurses, therapists, and physicians) about the applications of AI.

## Materials and methods

Study sample and setting

This cross-sectional study was conducted in Saudi Arabia between January 2023 and April 2023. The study was conducted among the healthcare providers of Saudi Arabia. The country has 485,688 healthcare providers working in primary health centers or specialized and private hospitals [10]. A simple random sampling was used to select healthcare providers from the different regions of Saudi Arabia to ensure respondents had an equal chance of selection, with them being recruited to fill out the survey. 

Sample size

The estimated sample size was 384 and determined using the formula ss = (Z2pq)/c2, where ss = sample size, Z = 1.96, p = 0.5, q = (1-p) = 0.5, and c = sampling error of 5%. In total, 1026 respondents participated. The selection criteria were as follows: inclusion criteria, any healthcare provider who worked in Saudi Arabia and consented to be part of the study; exclusion criteria, healthcare providers who did not consent to participate in the study or worked outside of Saudi Arabia.

Data collection tool

An online questionnaire was administered. A comprehensive literature review reviewed the studies on AI and healthcare providers’ readiness for its applications. We searched relevant papers in standard research databases such as PubMed, Science Direct, and Google Scholar (searching for ‘‘Assessment of readiness of healthcare providers of AI applications’’). We yielded some results about AI in the medical field but not much about the assessment of the readiness of healthcare providers in the applications of AI in Saudi Arabia. However, our search with the keywords, ‘‘AI, perceptions, healthcare,’’ yielded a relevant study by the Royal Free London NHS Foundation Trust. Their validated and structured questionnaire was used to collect responses [11]. The questionnaire was administered online through social media (Twitter, WhatsApp, Telegram) to reach the target population between January 2023 and March 2023. The participants were requested to participate after informed consent. The study's main objective was explained to them before sharing the questionnaire. The questionnaire comprised three parts: social demographic variables, perception, and worries.

Data analysis

The data set was analyzed using SPSS Statistics, version 26.0 (IBM Corp., Armonk, NY). Descriptive statistics and inferential statistics were conducted. The qualitative data was expressed as a percentage. The distribution of frequency was used to define each variable. The chi-square test was used to determine the association between different variables and to obtain the corresponding p-value. 

Ethical considerations

We obtained ethical approval from the permanent committee of the ethics of scientific research at Jouf University (Approval No. 1-07-44). Informed consent and confidentiality were some of the ethical considerations, with the assurance to respondents that they had the right to withdraw consent and abandon the process at any point during the survey.

## Results

A total of 1026 healthcare providers completed the questionnaires (Table 1). Of these, 539 (52.5%) were females and 487 (47.5%) were males. The majority of the participants (63.7%) were aged 25-35 years, while the minority (2.0%) were 65 years and above. Of the respondents, a vast majority (84.5%) were Saudi nationals, while 15.5% were non-Saudi nationals. The Saudi provinces represented by most participants were the central and northern regions, at 27.8% and 21.9%, respectively. The majority of the respondents were from the primary level (center healthcare), constituting 36.8%, while the secondary level (general hospital) constituted 36.2%. The majority of the participants (45.6%) were medical doctors. Additionally, the majority of the participants (46.5%) had 0-5 years of working experience, while the minority had more than 20 years of experience in healthcare facilities.

**Table 1:** Social demographic variables of respondents

Variables	Category	Frequency	Percentage
Gender	Female	539	52.5%
	Male	487	47.5%
Age	25-35 years	653	63.7%
	36-45 years	235	23.1%
	46-55 years	115	11.2%
	More than 65 years	21	2.0%
Nationality	Non-Saudi	159	15.5%
	Saudi	867	84.5%
The Saudi regions	Central province	286	27.8%
	Eastern province	151	14.7%%
	Northern province	225	21.9%
	Southern province	162	15.8%
	Western province	167	16.2%
	Others	35	3.6%
Level of healthcare	Primary (center healthcare)	378	36.8%
	Private hospital	116	11.3%
	Secondary (general hospital)	371	36.2%
	Tertiary (specialty hospital)	161	15.7%
Health profession	Manager	42	4.1%
	Medical doctor	468	45.6%
	Nurse	280	27.3%
	Other: open text	118	11.5%
	Physician associate	42	4.1%
	Therapist	76	7.4%
Working experience	0-5 years	477	46.5%
	5-10 years	269	26.2%
	10-15 years	156	15.2%
	15-20 years	70	6.8%
	More than 20 years	54	5.3%

Table 2 reports the perception of healthcare providers about AI. In this study, more than half (55.2%; 566) of the healthcare providers agreed that they had exposure to the term "artificial intelligence" during their studies in college. The majority of the participants (48.1%) were familiar with different applications of AI in their specialty, with (29.5%) having familiarity to some extent. More than half (62.8%) of the healthcare providers had encountered at least one application of AI in their work. The majority of the participants (57.9%) knew at least one term with regard to the difference between machine learning and deep learning. More than half (69.9%) of the participants noted that they had at one point in time used speech recognition or transcription application in their work. The majority of the respondents (74.6%) agreed that there could be serious privacy issues with the use of AI. More than half of the respondents (62.1%) agreed with the statement that “AI is more dangerous than nuclear weapons.” However, a vast majority (76.2%) of the participants decided that AI was useful in their area of study.

**Table 2:** Perception of healthcare providers about artificial intelligence (AI) * This statement is attributed to Elon Musk [11].

Item	Variable	Count	Percentage
Have you gotten exposed to the term artificial intelligence (AI) during your college study?	No	460	48.8%
	Yes	566	55.2%
Are you familiar with different applications of AI in your specialty?	No	230	22.4 %
	To some extent	303	29.5%
	Yes	493	48.1%
How many applications of AI have you come across in your work?	None	382	37.2%
	One	292	28.5%
	Two to four	292	28.5%
	More than four	60	5.8%
Do you know the difference between machine learning and deep learning?	Not at all	432	42.1%
	I only know one term	282	27.5%
	I know both terms, but the difference is not clear	214	20.9%
	I know both terms, and the difference is clear to me	98	9.5%
How often do you use speech recognition or transcription applications?	Never	309	30.1%
	Rarely	451	44.0%
	Weekly	169	16.5%
	On a daily basis	97	9.4%
Do you think there may be serious privacy issues with the use of AI?	Completely agree	302	29.4%
	Partially agree	464	45.2%
	Partially disagree	177	17.3%
	Completely disagree	83	8.1%
How much do you agree with the following statement, “AI is more dangerous than nuclear weapons”?*	Completely agree	287	28.0%
	Partially agree	350	34.1%
	Partially disagree	213	20.8%
	Completely disagree	176	17.1%
How useful do you think AI could be in your area of work?	Extremely useful	350	34.1%
	Useful	432	42.1%
	Of limited use	201	19.6%
	Of no use at all	43	4.2%

Table 3 presents the worries of healthcare providers about AI. In this study, the majority (73.3%; 752) of the healthcare providers were worried that AI would replace them at their job. A majority (76.2%) of the respondents supported initiatives to integrate AI courses into the medical curriculum. The majority of the respondents suggested the first and second years, constituting 33.3% and 21.7% of the participants, respectively, to be suitable years for health professional students to be taught about AI. A vast majority (84.9%) of the participants agreed that collaboration between medical schools with engineering and computer science faculties could be a game changer to provide a road for incorporating AI into the medical curriculum.

**Table 3:** Worries of healthcare providers about artificial intelligence (AI)

Item	Variable	Count	Percentage
How worried are you that artificial intelligence (AI) will replace you at your job?	Extremely worried	244	23.8%
	Moderately worried	312	30.4%
	Mildly worried	196	19.1%
	Not worried at all	274	26.7%
Are you with or against integrating AI courses into the medical curriculum?	Agree	782	76.2%
	Disagree	244	23.8%
In your opinion, what is a suitable year to teach health professions students about AI?	First	342	33.3%
	Second	223	21.7%
	Third	195	19.0%
	Forth	141	13.7%
	Fifth	125	12.3%
‏Can collaboration between medical schools with engineering and computer science faculties be a game changer to provide a road for incorporating AI in the medical curriculum?	Agree	871	84.9%
	Disagree	155	15.1%

Table 4 presents the mean positive perception score at 37.6 (SD=8.41; range 0-241). The mean positive perception score was significantly associated with age (p=0.021), level of healthcare (p=0.031), health profession (p=0.041), and working experience (p=0.026). The gender of the participants (p=0.842), nationality (p=0.715), and the Saudi regions (p=0.065) were found to have no significant association with the mean positive perception score.

**Table 4:** Association between the mean positive perception score of AI and the demographics of participants AI: Artificial intelligence

Variables	Participants' characteristics	Mean	Std	F-value	t-value	P-value
Gender	Female	8.62	1.52	-	0.013	0.842
	Male	8.07	1.41			
Age	25-35 years	8.68	1.95			0.021
	36-45 years	8.76	1.68			
	46-55 years	8.12	1.34			
	More than 65 years	8.53	1.72	3.721	-	
Nationality	Non-Saudi	8.34	1.05		0.312	0.715
	Saudi	8.02	1.82			
The Saudi regions	Central province	8.29	1.05			0.065
	Eastern province	7.97	1.83			
	Northern province	8.82	1.73			
	Southern province	8.42	1.90		0.023	
	Western province	7.82	1.89			
	Others	8.81	1.33			
Level of healthcare	Primary (center healthcare)	8.54	1.64			0.032
	Private hospital	7.98	1.83			
	Secondary (general hospital)	8.05	1.78	3.751		
	Tertiary (specialty hospital)	8.10	1.72			
Health profession	Manager	8.83	1.82			0.041
	Medical doctor	8.91	1.06	3.752		
	Nurse	8.32	1.19			
	Other: open text	8.21	1.62			
	Physician associate	8.62	1.89			
	Therapist	8.61	1.83			
Working experience	0-5 years	8.72	1.16			0.026
	5-10 years	8.84	1.54	3.459		
	10-15 years	8.43	1.62			
	15-20 years	8.36	1.83			
	More than 20 years	8.45	1.45			

## Discussion

This study’s main objective was to determine the perception and worries of Saudi healthcare providers about the applications of AI in Saudi healthcare facilities. The results of this study indicated that more than half (55.2%) of the respondents had good knowledge of AI, with 48.1% of them being familiar with the application of AI in their specialty. A good proportion of the participants (57.9%) knew at least one term with regard to the difference between machine learning and deep learning. More than half (69.9%) of the participants indicated that they had at one point in time used speech recognition or transcription application in their work. While a significant proportion of the respondents agreed that there may be serious privacy issues with the use of AI, a vast majority (76.2%) of the participants agreed that AI was useful in their area of study. This finding is consistent with the study conducted in the United States by Liu et al. (2022), which reported that 91.5% of medical students agreed that training in AI during medical school would be useful for their future [12]. Despite the fact that most respondents agreed that the use of AI could cause serious privacy issues, a large proportion of respondents were interested in learning AI in order to increase efficiency in performance and career growth. The findings corroborate the study conducted in Canada by Teng et al. (2022), which found that 57.3% of medical students believed that AI would make the healthcare profession better and would have a positive impact on their careers [13].

Although in this study 73.3% of the healthcare providers believed that AI would replace them at their job, Jha et al.'s (2022) study revealed that over half of the respondents agreed that AI would reduce the number of jobs for healthcare professionals [14]. Similarly, Ahmed et al.'s (2022) study revealed that approximately 70% of medical students and 81.8% of doctors from the study population acknowledged that AI could serve as a practitioner’s aid soon, and most of them did not consider AI as a physician’s replacement but rather a physician’s diagnostic tool [15]. A majority (76.2%) of the respondents of this study were in support of initiatives aimed at integrating AI courses into the medical curriculum. The findings are consistent with the study conducted by Khanagar et al. (2021), which found that more than half of dental students in Riyadh, Saudi Arabia supported the usage of AI in dentistry [16]. A vast majority (84.9%) of the participants of this study agreed that collaboration between medical schools with engineering and computer science faculties could be a game changer to provide a road for incorporating AI into medical curricula. The finding is consistent with Singh et al.'s (2020) assertion that enhanced integration of real-world artificial intelligence coupled with modern technological innovations in healthcare systems would go a long way in increasing efficiency and effectiveness in service delivery across healthcare facilities [17].

The mean perception of AI in this study was 37.6 (SD=8.41; range 0-241). Age, level of health, health profession, and working experience all had a significant impact on the positive perception score (p=0.021; p=0.031; p=0.041; p=0.026). However, there was no significant association between gender, nationality, and Saudi regions with a mean positive perception score. This could be better explained by the fact that experienced healthcare professionals demonstrate a higher level of knowledge and awareness than juniors. Furthermore, prior exposure to AI during seminars and training may have influenced this situation. This study would add a significant contribution to the implementation of AI and would serve as a reference for future studies. The findings could also be used by educational and healthcare institutions to develop appropriate training to improve AI courses.

The results of this study may have been predisposed to some limitations. A cross-sectional study of this nature has a limitation attributed to its inability to assess causal relationships. Additionally, considering that the study involved the administration of online questionnaires, the study relied on respondents accurately documenting their responses without the ability to check this, which may have contributed to a potential bias. Despite these limitations, the researcher believes that the study provides useful insights into healthcare providers’ perspectives on the integration and use of AI in healthcare facilities in Saudi Arabia.

## Conclusions

The findings in this study indicated a positive perception of AI among Saudi healthcare providers. Even though a substantial majority of Saudi healthcare providers were worried that AI would replace their jobs, the study revealed that AI serves as a crucial practitioner’s tool rather than a physician’s replacement. Looking ahead, it is likely that AI will continue to play a significant role in shaping the future of healthcare in Saudi Arabia. The government and healthcare institutions need to invest in AI research, development, and education to foster innovation and ensure that healthcare professionals are equipped with the necessary skills to leverage AI effectively and ethically. Furthermore, addressing the challenges related to data privacy, security, and regulations will be crucial to maximize the benefits of AI in Saudi health facilities while safeguarding patient rights and well-being.
